# Evolutionary constraints on yeast protein size

**DOI:** 10.1186/1471-2148-6-61

**Published:** 2006-08-15

**Authors:** Jonas Warringer, Anders Blomberg

**Affiliations:** 1 Department of Cell and Molecular Biology, Lundberg Laboratory, Göteborg University Medicinaregatan 9c, 41390 Göteborg, Sweden

## Abstract

**Background:**

Despite a strong evolutionary pressure to reduce genome size, proteins vary in length over a surprisingly wide range also in very compact genomes. Here we investigated the evolutionary forces that act on protein size in the yeast *Saccharomyces cerevisiae *utilizing a system-wide bioinformatics approach. Data on yeast protein size was compared to global experimental data on protein expression, phenotypic pleiotropy, protein-protein interactions, protein evolutionary rate and biochemical classification.

**Results:**

Comparing the experimentally determined abundance of individual proteins, highly expressed proteins were found to be consistently smaller than lowly expressed proteins, in accordance with the biosynthetic cost minimization hypothesis. Yeast proteins able to maintain a high expression level despite a large size tended to belong to a very distinct set of protein families, notably nuclear transport and translation initiation/elongation. Large proteins have significantly more protein-protein interactions than small proteins, suggesting that a requirement for multiple interaction domains may constitute a positive selective pressure for large protein size in yeast. The higher frequency of protein-protein interactions in large proteins was not accompanied by a higher phenotypic pleiotropy. Hence, the increase in interactions may not reflect an increase in function differentiation. Proteins of different sizes also evolved at similar rates. Finally, whereas the biological process involved was found to have little influence on protein size the biochemical activity exerted by the protein represented a dominant factor. More than one third of all biochemical activity classes were enriched in one or more size intervals.

**Conclusion:**

In yeast, there is an inverse relationship between protein size and protein expression such that highly expressed proteins tend to be of smaller size. Also, protein size is moderately affected by protein connectivity and strongly affected by biochemical activity. Phenotypic pleiotropy does not seem to affect protein size.

## Background

One of the more surprising observations in the early genome studies was the enormous variation in genome size, not only among eukaryotes in general (>200,000 fold variation), but also within kingdoms (e.g. plants, >1,000 fold variation) [1]. Even among closely related species, genome size has been found to exhibit remarkably large variation [2]. Nevertheless, the evolutionary significance of this variation is still unknown. Given that the number of genes varies much less than overall genome size (e.g. only 5-fold between yeast and humans) scientific focus has been on the intergenic DNA that makes up the bulk of most eukaryotic genomes. Several hypothesizes has also been put forward to explain the variation in the size of intergenic DNA, ranging from the notion that the unnecessary "junk" DNA is not really unnecessary at all [3] to the suggestion that the evolutionary cost of carrying junk DNA is so minimal that the negative selective consequences may be disregarded. The latter hypothesis stems from the observation that much of the junk DNA is selfish in nature [4,5] making it more likely that its accumulation has little to do with the fitness of the organism itself [2]. Currently, it is becoming increasingly apparent that a large genome size constitutes a real and considerable burden. A large genome size tends to correlate with delayed mitotic and meiotic division [6-8] decreased plant invasiveness of disturbed sites [9] lower maximum photosynthetic rates in plants [2] and lower metabolic rates in mammals [10] and birds [11,12]. Furthermore, genera with large genome sizes tend to contain fewer species and species with large genomes tend to be underrepresented in harsh environments [2]. These observations suggest that genome size minimization constitute a dominant selective force.

In lower organisms such as yeast where intergenic DNA comprise less than 30% of the genome [13] – as opposed to 98% in human [14] – it may be argued that reducing the size of coding DNA significantly affects genome size. Thus, in lower organisms minimizing protein size would enable a higher cell division rate and result in lower DNA maintenance costs. In addition it has been suggested [1] that a reduction in protein size vastly reduces protein biosynthetic costs, directly by decreasing the energetic costs of translation [15] and indirectly by reducing the cost of chaperones required to fold large multi-domain proteins [16]. Indeed, gene length in eukaryotes tends to correlate negatively with synonymous codon usage bias [17-20], a tentative measure of protein expression levels. In addition, proteins with a high synonymous codon usage bias tend to preferentially contain amino acids that are less energetically costly [21], a factor essentially determined by amino acid weight [22]. Thus, a requirement for high protein expression may impose a biosynthetic cost constraint on protein size.

Despite the seeming fitness benefits of minimizing protein size, the size of individual proteins within a genome displays as remarkable a variation as the size of genomes within a kingdom; for example in *S. cerevisiae*, the protein size range spans over two orders of magnitude; from 25 to more than 4.100 amino acids. Thus, strong selective forces counterbalance the evolutionary pressure to minimize protein size. In this article we considered four hypotheses regarding the nature of the selective forces that favor a large protein size: i) Larger proteins are involved in multiple biological processes, therefore requiring multiple functional domains. This may be reflected in a higher extent of phenotypic pleiotropy among large proteins. ii) Larger proteins need to be more interconnected in the protein-protein network and thus may contain more protein-protein interaction domains. iii) The size requirements of individual functional domains may infer vastly different size constrains on different classes of proteins, i.e. large and small proteins would tend to exert very different biochemical activities in the cell and have differing function annotations. iv) Large proteins are more robust to changes in amino acid composition and may tolerate a higher mutation rate without loss of function.

These hypotheses were considered using the *S. cerevisiae *genome which has been re-annotated [13,23] and is essentially definite with regards to protein size annotation and for which there is ample genome-wide, experimental data on available.

## Results and discussion

### Smaller proteins are more abundant than larger proteins

**Figure 1:**
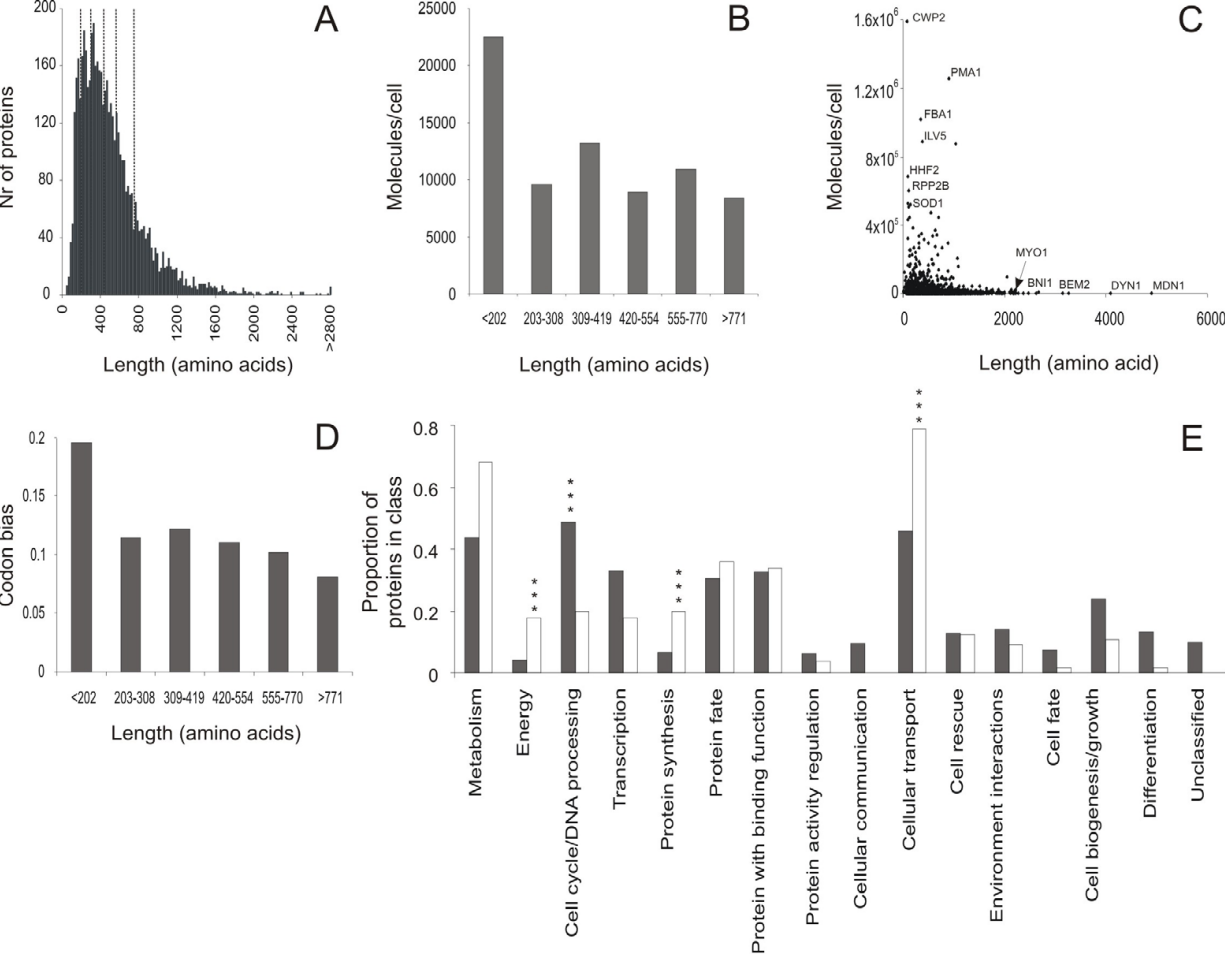
**Smaller proteins are more highly expressed than larger proteins**. Comparing *S. cerevisiae *protein expression and protein size data. A) Size distribution of re-annotated yeast protein lengths (average of 501 amino acids). Dashed lines indicate limits for size categories. B) Experimentally determined mean protein abundance (molecules/cell) in different (bins containing equal numbers of proteins) size categories during exponential growth [24]. C) Comparing protein abundance (molecules/cell) and protein length for individual proteins. Outliers are indicated. D) Mean codon bias in different (bins containing equal numbers of proteins) size categories during exponential growth. E) Proportion of both highly expressed (>12,273 molecules/cell) and (length>771) large proteins (empty bars) as compared to large proteins in general (filled bars) in different functional classes.

A negative correlation has been reported in eukaryotes between codon usage [17-20] and protein size as well as between frequencies of amino acids usage and their biosynthetic costs [21,22]. Thus, evolutionary constraints may reduce the size of heavily expressed proteins, thereby minimizing biosynthetic costs of protein translation and folding. However, in prokaryotes such as *Escherichia coli *codon usage correlate positively with protein size [17], indicating that this assumption is not necessarily true. Codon usage is, however, only a tentative indicator of protein expression. A more precise measure of protein expression is provided by experimental quantification of the abundance of individual proteins, such as has been performed in *S. cerevisiae *for a largely complete set of encoded proteins [24]. To investigate the correlation between protein expression and size in *S. cerevisiae *we compared recently re-annotated yeast protein lengths to data on protein abundance (molecules/cell) during exponential growth in optimal conditions [24]. Overall, there is a highly significant negative correlation (Spearman rank = -0.16, p = 1.6E-23) clearly demonstrating that larger proteins tend be less abundant than smaller proteins. However, dividing proteins according to their length into equally sized bins (Fig 1A) and comparing the average protein abundance within each bin, the correlation between protein size and expression appears to be of unequal strength in different size intervals (Fig 1B). In fact, only the smallest proteins (length<202 amino acids) proteins deviated in a highly significant manner (Mann-Whitney, p = 0.0007) from proteins in general; on average, the smallest proteins were twice as abundant as the average protein. The strong contribution of the very small proteins to the overall correlation is also evident from a plot of protein abundance versus protein size for individual proteins (Fig 1C). Also considering codon bias as a tentative measure of protein expression the smallest proteins appear as most strongly affected by the correlation between protein size and expression (Fig 1D). Hence, in yeast, minimizing biosynthetic costs by reducing the size of highly expressed proteins constitutes a favorable evolutionary strategy primarily for the very small proteins. This is hardly surprising as the smallest proteins contain a disproportionably high frequency of ribosomal proteins which account for a large fraction of the total costs of protein production (see below). It should be noted however, that even excluding the class with the smallest proteins (<202 amino acids) there is a significant negative correlation between protein size and expression in yeast (Spearman rank = -0.15, p = 3.2E-21).

In the light of the selective pressure to minimize biosynthetic cots by reducing the size of highly expressed proteins we reason that proteins that are expressed to high levels despite a large size may be especially interesting from a biological function perspective. Comparing the 56 proteins that are both highly expressed (above the overall average of 12,273 molecules/cell) and large (length>771 amino acids) to all large proteins we find that these proteins are especially prone (hypergeometric distribution assumption, p < 0.001) to be involved in protein synthesis, energy metabolism, and cellular transport (Fig 1E). Notably, three translation initiation factors, Fun12p, Clu1p and Rpg1p as well as three translation elongation factors, Eft1p, Eft2p andYef3p are both large and highly expressed. It may also be noted that the enrichment of cellular transport functions include four of the eight components of the COPI coatomer vesicle complex, Sec21p, Sec26p, Sec27p and Cop1p as well as a high proportion of nuclear transport function genes, both mRNA export and protein import.

### Protein connectivity affects protein size in yeast

**Figure 2:**
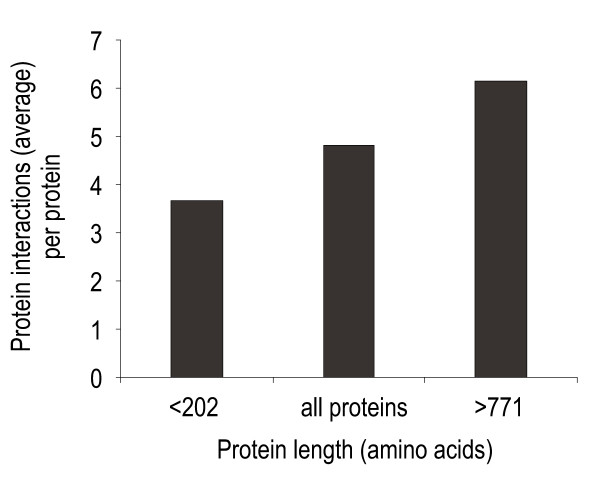
**Large protein size is partially maintained by a demand for higher connectivity**. Comparing *S. cerevisiae *protein-protein interaction data and protein size. Average number of protein-protein interactions in different size categories is displayed.

We hypothesized that the evolutionary pressure that maintains large protein size may reflect an underlying selection for more protein-protein interaction domains. In accordance with this hypothesis, we expected large proteins to display higher connectivity in the physical protein-protein interaction network than small proteins, i.e. they should participate in more protein-protein interactions. Using available protein-protein interaction data from yeast 2-hybrid and protein affinity precipitation studies we compared the average connectivity for proteins in different size intervals. No statistically significant correlation between protein size and connectivity could be observed for proteins of intermediate size. However, comparing the size extremes, the largest proteins (>771 amino acids) entertained on average twice the number of protein-protein interactions as the smallest proteins (<202 amino acids) (Fig 2). This difference was highly significant (Students t-test, p = 2.1 × 10^-6^). To ensure that this correlation between protein connectivity and protein size was not influenced by the above-reported stronger correlation between protein abundance and protein size, partial correlation analysis, controlling for protein abundance, was carried out. However, controlling for protein abundance did not substantially affect the correlation between connectivity and size (partial correlation, p = 2.6 × 10^-6^).

We conclude that the selective pressure to maintain a large protein size at least partially may be a selective pressure to entertain more protein-protein interactions.

### Multi-functionality does not favor a large protein size

**Figure 3:**
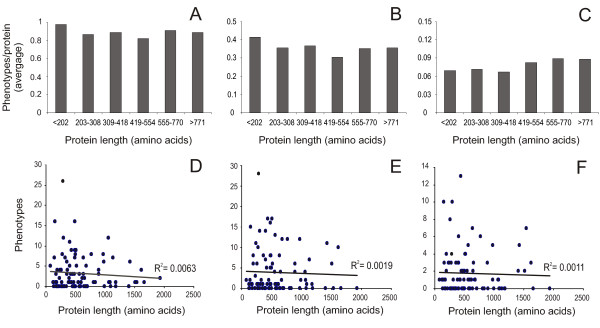
**Multi-functionality does not favor large protein size**. Comparing quantitative phenotypes of *S. cerevisiae *deletion strains [27] and protein size A-C) average number of significant (p < 0.001) phenotypes (LPI) in different size categories; data represent all viable deletion strains cultivated in five different conditions (see Material and methods) D-F) number of significant (p < 0.001) phenotypes (LPI) versus protein size for 96 deletion strains cultivated in 36 different conditions (see Material and methods), linear correlation r^2 ^indicated A, D) adaptation time B, E) growth rate C, F) growth efficiency.

Multi-functionality in individual proteins requires the maintenance of multiple biochemical domains; hence multi-functionality may be regarded as a possible selective force favoring large protein sizes in the face of the evolutionary pressure to reduce genome size and biosynthetic costs. It is reasonable to expect that yeast protein multi-functionality at least partially is reflected in the number of phenotypes displayed by yeast knockout strains, i.e. multi-functional proteins should, on average, be more pleiotropic than mono-functional proteins [25]. Hence, in accordance with the hypothesis of a correlation between multi-functionality and large protein size, we would expect knockout strains deleted for large proteins to display higher pleiotrophy, i.e. more phenotypes, than individuals lacking smaller proteins. The deletion of essentially every *S. cerevisiae *open reading frame has been completed [26], enabling the evaluation of this hypothesis. We have earlier introduced an approach for the precise quantification of phenotype/fitness changes in yeast by automated micro-Cultivation of isogenic populations [27]. Applying the methodology on a genome-wide scale we obtained exact measures of gene-by-environment interactions, termed Logarithmic Phenotypic Indexes (LPI), for each non-essential yeast protein. To evaluate the multi-functionality hypothesis regarding protein size we correlated the phenotypic behavior of each deletion strain during five different growth conditions (see Material and methods) and using three fitness measures, time to initiate reproduction (lag-phase), rate of reproduction during exponential growth (generation time) and efficiency of reproduction (population density reached) to the size of the deleted proteins. Slightly surprisingly, we found no correlation between the number of phenotypes and the size of the deleted proteins (Fig 3A–C). This lack of correlation was evident regardless of which fitness measure was considered.

To account for the possibility that the lack of correlation arises from the use of a limited and biased number of growth conditions we performed in depth phenotypic profiling data for 96 deletion strains, randomly selected with regards to protein size, during 36 very diverse growth conditions. This data was further compared to the size of each deleted protein. However, for none of the fitness measures investigated, adaptation time, growth rate and growth efficiency, did we find a significant difference in the number of significant phenotypes between large and small proteins (Fig 3D–F); neither did we find any significant correlation between protein size and the level of protein dispensability as the magnitude of phenotypes (LPI) were similar for large and small proteins (data not shown). The selective pressure to maintain protein size therefore does not appear to be a selective pressure for pleiotrophy/multi-functionality within individual proteins.

### Proteins of different sizes evolve at similar rates

Using a limited set of 31 *Drosophila melanogaster *proteins Seligmann observed that amino acid weight minimization, i.e. the selective pressure to reduce the number of heavy amino acids in large proteins, affected the rate of amino acid replacements [22]. Our final hypothesis raised the possibility that large proteins are more robust to changes in amino acid composition and may tolerate a higher mutation rate without loss of protein function simply because of their size. To investigate whether large proteins in yeast are more tolerant to mutations and hence evolve at a higher rate we correlated protein size data to data on the rate of individual changes of base pairs within proteins as represented by the ratio of amino acids changing mutations versus silent mutations (dN/dS) [28]. We found no significant correlation between dN/dS ratios (linear correlation, r^2 ^= E-6) and protein size in yeast. Protein evolutionary rate is known to be strongly influenced by protein expression level [29], however, even controlling for this variable (protein absolute abundance) no correlation was found between evolutionary rate and protein size (partial correlation, p = 0.15).

We conclude that the evolutionary rate does not constitute a selective force that substantially constrains protein size.

### Protein size is constrained by the size requirements of the biochemical domain

**Figure 4:**
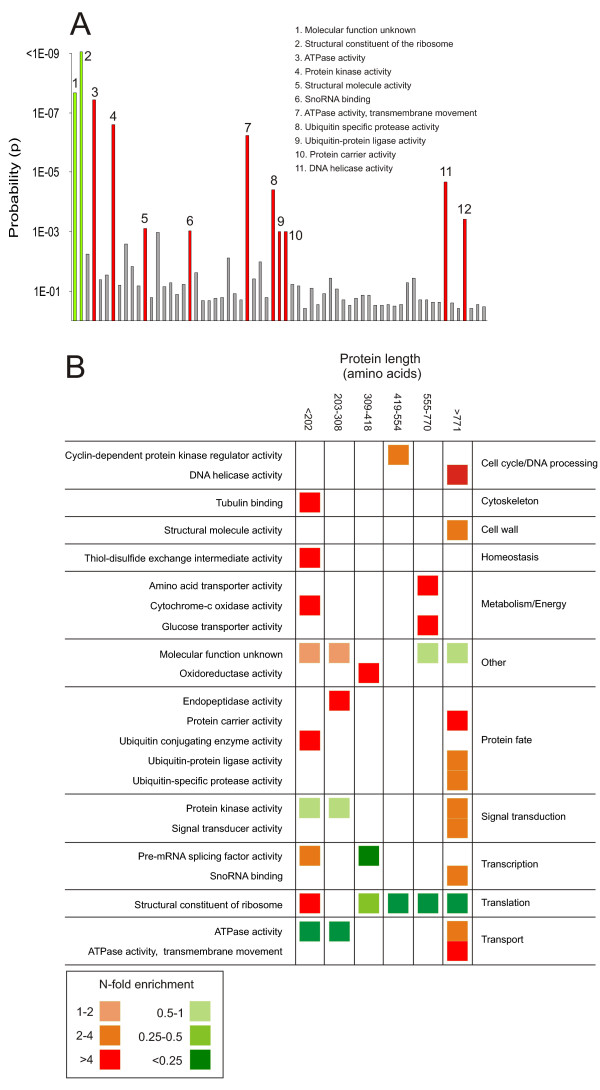
**Protein size is constrained by the size requirements of the functional domain**. Comparing *S. cerevisiae *biochemical activity annotation data and protein size A) Biochemical activity classes with a disproportionate representation among the largest proteins (length>771 amino acids). Probability (p) of a deviation from the representation among all proteins is displayed (hypergeometric distribution). Significant (p < 0.001) deviations are indicated; red = overrepresentation, green = underrepresentation. B) Biochemical activity classes with a deviating (p < 0.001) representation in any size interval. N-fold overrepresentation (red) and underrepresentation (green) as compared to the expected representation are indicated.

In an idealized situation the length of a protein would be completely dependent on its function and the variance in the lengths of an organism's proteins would reflect the diversity of functions in the particular organism [30]. To evaluate to what extent protein function in practice influence protein length, we studied the frequency of different biochemical activities among yeast proteins in different size intervals and compared to the corresponding frequency among all yeast proteins (Fig 4A). The influence of function on protein size was found to be strong. Of 65 investigated biochemical activities 22 were highly enriched (p < 0.001) in at least one size interval (Fig 4B). Hence, more than one third of the investigated biochemical activities displayed an uneven distribution with regards to protein size. Not surprisingly the enrichments were most numerous among the size extremes; seven biochemical activities were significantly overrepresented among the smallest proteins whereas ten were significantly overrepresented among the largest (Fig 4B). The biochemical activities enriched among the largest proteins included several broad categories involved in signal transmission, notably protein kinase activity and signal transducer activity (Fig 4A, B). Few paralogous proteins were found within these member-rich categories, indicating that the overrepresentations constitute true functional enrichments and not artifacts of extensive gene duplication. Also among the smallest proteins several of the enriched categories, such as tubulin binding and protein transporter activity (Fig 4B), were so diverse with regards to the evolutionary history of the proteins that widespread sequence similarity could be ruled out as a cause of the overrepresentations. In some, cases the skewness of the function distributions was extreme, e.g. for protein kinases where 24 out of 53 proteins were found in the largest size category, but none in the smallest. Similarly, of the 203 proteins annotated as having a biochemical activity as structural constituents of the ribosome we found 137 among the smallest proteins but none among the largest proteins.

To ascertain that the observed correlation between biochemical activity and protein size was not an artifact arising from an underlying correlation between biological process and protein size, we also analyzed the frequency distribution of biological process annotation data with regards to protein size. Of 299 analyzed biological processes none were highly overrepresented (p < 0.001) among either large or small proteins (data not shown). A clear example of that it is the biochemical activity rather than the biological process that forms the correlation between protein function and protein length is provided by proteins involved in ubiquitin mediated proteolysis. Components of this biological process were evenly distributed with regards to protein size. However, on the level of biochemical activity both ubiquitin protein ligase activity and ubiquitin specific protease activity were enriched among the largest proteins whereas ubiquitin conjugating activity was highly enriched among the smallest proteins (Fig 4B). We conclude that the nature of the biochemical activity exerted by a protein constitute a dominant selective pressure to maintain large protein size.

## Conclusion

Using experimental data from a global *S. cerevisiae *study on protein abundance [24], we here demonstrated that smaller proteins tended to be more highly expressed than larger proteins. Our observations are in line with reports of a negative correlation between codon usage and protein size in eukaryotes [17-19] as well as with observations that large proteins tend to contain energetically less costly amino acids [21,22]. Also, it has been reported that mRNA abundance restricts maximum protein length [31]. Taken together, these observations strongly suggest that the biosynthetic cost minimization hypothesis is biologically relevant.

The correlation between protein size and expression was found to be strongest for the smallest and most expressed proteins, which include a disproportionably high frequency of ribosomal proteins. In a rapidly growing yeast cell as much as 50% of RNA polymerase II transcription is devoted to ribosomal proteins [32]; thus, the biosynthetic cost constraints that limit the size of highly expressed proteins are expected be extremely strong for these proteins.

It should be observed that the here presented correlation between protein length and low protein abundance does not allow for a clear determination of cause/effect relationships. In fact the two alternatives – i.e. protein size acting as evolutionary constraint on protein expression and protein expression restricting protein size – are not mutually exclusive and may reflect parallel selective forces.

Additionally, we investigated the nature of the evolutionary forces that maintain the size of large proteins despite the selective pressure to minimize DNA maintenance/replication times as well as biosynthetic costs. Genetic pleiotropy, i.e. the ability of a mutation in a single gene to give rise to multiple phenotypic outcomes [33], has been shown to be surprisingly wide-spread in yeast and to correlate to a variety of protein features, such as function and chromosomal position [25,34]. Although a single function may have multiple phenotypic outcomes, pleiotropy may be argued to be at least a vague indicator of the degree of multi-functionality. We hypothesized that if there is a general requirement for multi-functionality in large proteins, thus imposing evolutionary constrains on size, we expected to see some sort of correlation between the degree of pleiotropy and protein size. However, no such correlation was found, providing tentative indications that large proteins in general do not possess more functions than smaller proteins.

We also investigated a possible selective pressure for more protein-protein interactions, requiring multiple interaction domains, in large proteins. Protein connectivity is widely known to affect the functional importance of proteins [35] which in turn is known to correlate positively with protein size [30], supporting the plausibility of such a hypothesis. Mining available 2-hybrid and protein affinity precipitation data, we found larger proteins to have significantly more interaction partners than smaller proteins. Thus, a requirement for multiple interaction domains may be considered to act as a balancing selective force, partially offsetting the general fitness benefit of minimizing protein size. This higher connectivity does not transform into higher pleiotrophy. One possible explanation of this seeming anomaly is that the more frequent protein-protein interactions in large proteins may reflect a specific increase in input connectivity. In other words, large proteins would be subject to more regulatory signals but would not have more functional targets.

It is tempting to interpret the correlation between protein size and a high number of protein interactions as a demand for a larger protein size in proteins whose functions require a high connectivity. However, the here presented correlation does not allow for such a strict assignment of evolutionary cause/effect relationship. It cannot be excluded that proteins of larger size are more prone to form protein-protein interactions and, hence, that increasing protein size drives connectivity.

In the idealized situation of a total absence of general constraints on protein size, the length of an individual protein would be completely dependent on the size requirements of its domains. However, in the non-idealized reality the extent to which function balances the different general constraints and determines protein size is unknown. It has been observed that proteins with conserved and essential functions tend to be longer than proteins with highly less conserved and non-essential functions [30]. We here show that the individual protein function constitutes a dominant factor in the determination of protein size in yeast. Interestingly, it was found that it is the actual biochemical activity exerted by the protein, rather than the biological process involved, that is crucial. Not a single protein-size dependent enrichment was observed for different biological processes whereas one third of the investigated biochemical activities were highly overrepresented among either the smallest or the largest proteins. This strongly suggests that it is the size requirements of the individual biochemical domains that impose the strict limits on protein size. Some of the biochemical activity categories here revealed to contain disproportionably many large proteins, notably protein kinases and transcription factors had earlier been noted to produce above average-sized transcripts [36]. It should be noted that, using much broader definitions of biological processes than the here applied, Brocchieri *et al*. showed that proteins involved in "metabolism" and "cellular processes" tended to be longer than expected [37]. The here reported strong correlation between size and functional variability among yeast proteins probably reflects the underlying size requirements imposed by different structure motifs. Such an assumption is supported by the observation that it is biochemical activity rather than biological process which correlates to protein size. It is well established that proteins with similar biochemical activities share extensive structure similarities whereas few such correlations have been reported among proteins involved in the same cellular pathways.

## Methods

### Protein size data

A complete set of *S. cerevisiae *genes was obtained from the Saccharomyces Genome Database [38]. To avoid infiltration from dubious open reading frames and to decrease statistical noise data was filtered according to Kellis et al [13]; dubious genes not conserved between closely related yeast species were thus discarded (5256 genes were retained). Protein size was here considered as protein length (number of amino acids), however, as the correlation between protein length and protein weight in yeast is essentially linear (r^2 ^= 0.9987) protein length and weight may be regarded as equivalent measures.

### Protein expression data

To investigate whether the reported negative correlation between codon bias and protein size reflects a true evolutionary constraint by protein size on protein expression, protein size data (as above) was compared to data on protein abundance (molecules/cell) obtained by Ghaemmaghami et al [24]. The comparison encompassed 3663 epitope-tagged open reading frames expressed from their natural chromosomal locus during exponential growth in optimal conditions.

### Phenotypic data

To investigate whether the demand for increased protein size represents a demand for multiple functional domains and pleiotrophy, protein size data (as above) was compared to quantitative data on the phenotypes of haploid deletion strains cultivated in isolation. To avoid possible biases arising from the use of either a limited number of growth conditions or a limited number of deletion strains two separate sets of phenotypic data was used [27,39]: i) phenotypic data on 96 deletion strains, randomly chosen with regards to protein size and cultivated in 40 diverse conditions of environmental stress ii) phenotypic data on all 4,220 deletion strains cultivated in optimal conditions as well as during four conditions of environmental stress – sodium chloride (salt stress), paraquat (superoxid anion production), diamide (elevated oxidation levels) and DTT (decreased oxidation levels). Strain- and environment normalized phenotypes (Logarithmic Phenotypic Indexes – LPI) reflecting genuine strain-by-environment interactions were used in both comparisons. Analyses using Logarithmic Strain Coefficient, LSC, data not normalized to the growth behavior of the knockout strain in non-stressed conditions yielded similar results (data not shown). Furthermore, to avoid possible biases arising from the use of phenotypic data representing a single component of fitness three distinct fitness indicators were used: i) time to adapt to the environmental stress (lag phase) ii) rate of reproduction during exponential growth and efficiency of growth (population density reached). Phenotypic data for all genes in question can be accessed at the Prophecy database [40].

### Evolutionary rate/duplication data

To investigate the possibility of a correlation between protein size and the rate of individual protein evolution protein size data (as above) was compared to data dN/dS and dN/dS' ratios taken from Wall et al [28]. The comparison comprised all genes conserved between four closely related species of the *Saccharomyces sensu stricto *group [13], excluding frame shifted or intron containing open reading frames, for a total of 2,918 genes.

### Interaction data

To investigate whether the demand for increased protein size represents a demand for multiple protein-protein interaction domains, protein size data (as above) was compared to protein-protein interaction data obtained from the GRID database [41] encompassing several large scale yeast 2-hybrid and affinity precipitation studies as well as numerous small scale investigations. Protein-protein interaction data, corresponding to 25,215 interactions, was obtained for the 5,256 genes.

### Functional classification data

To investigate whether the demand for increased protein size represents a demand for certain large biochemical domains, i.e. if certain biochemical functions are overrepresented among proteins of larger size, protein size data (as above) was compared to the GO biochemical activity classification data obtained from SGD [38]. For each biochemical activity (total of 65 activities) the frequency in each protein size category was compared to the frequency among all proteins included in the study. Significant overrepresentations were determined assuming a hypergeometric data distribution. To account for the possibility of extensive sequence similarity causing the observed overrepresentation, an additional functional enrichment analysis was carried out excluding all paralogous yeast proteins. Yeast sequence paralogs were defined as yeast proteins with a (Blastp) sequence similarity (e-value) to another yeast protein of less than 10^-10 ^over at least 50% of the coding sequence. With the exception of ribosomal proteins, the exclusion of paralogous proteins did not substantially affect the enrichment of specific functional classes. Protein size data was also compared to biological process classification data obtained from MIPS [42] in a similar manner.

## Authors' contributions

JW designed and carried out the study, performed the statistical analysis and drafted the manuscript. AB participated in its design and coordination and helped to draft the manuscript. Both authors read and approved the final manuscript.
